# Correlated Genetic and Ecological Diversification in a Widespread Southern African Horseshoe Bat

**DOI:** 10.1371/journal.pone.0031946

**Published:** 2012-02-27

**Authors:** Samantha Stoffberg, M. Corrie Schoeman, Conrad A. Matthee

**Affiliations:** 1 Evolutionary Genomics Group, Department of Botany and Zoology, Stellenbosch University, Stellenbosch, Republic of South Africa; 2 School of Life Science, Westville Campus, University of KwaZulu-Natal, Republic of South Africa; University of Western Ontario, Canada

## Abstract

The analysis of molecular data within a historical biogeographical framework, coupled with ecological characteristics can provide insight into the processes driving diversification. Here we assess the genetic and ecological diversity within a widespread horseshoe bat *Rhinolophus clivosus* sensu lato with specific emphasis on the southern African representatives which, although not currently recognized, were previously described as a separate species *R. geoffroyi* comprising four subspecies. Sequence divergence estimates of the mtDNA control region show that the southern African representatives of *R. clivosus* s.l. are as distinct from samples further north in Africa than they are from *R. ferrumequinum*, the sister-species to *R. clivosus*. Within South Africa, five genetically supported geographic groups exist and these groups are corroborated by echolocation and wing morphology data. The groups loosely correspond to the distributions of the previously defined subspecies and Maxent modelling shows a strong correlation between the detected groups and ecoregions. Based on molecular clock calibrations, it is evident that climatic cycling and related vegetation changes during the Quaternary may have facilitated diversification both genetically and ecologically.

## Introduction

The fields of ecology and conservation biology centre around the interactions between biological diversity and the environment. Species are arguably the most important taxonomic unit used when testing broad-scale ecological patterns. Knowing the number and uniqueness of species, is important for conservation planning as it reflects species richness, endemism as well as potential threats of losing biodiversity [1]. In recent years the newly discovered mammal species account for approximately 10% of what were known prior to 1993, with higher than expected numbers in some orders, particularly the Chiroptera [2], [3]. In the past, the trend of increasing numbers of species has been attributed to “taxonomic inflation” [1], although others argue that it reflects the underlying nature of species [4] and highlight the importance of evaluating previously unrecognized biodiversity (including genetic diversity) and the implications of this for ecological studies, conservation planning and the preservation of ecosystem services [3], [5].

DNA-based studies enable the analysis of genetic diversity within taxa and in particular the quantification and subsequent recognition of cryptic and sibling species [6]. Often, many species shown to be complexes harbouring cryptic species do not demonstrate morphological differences either due to nonvisual mating signals (e.g. sound) or selection that favours morphological stasis [5]. Within bats, substantial difficulties are associated with species identification as exemplified by the high incidence of cryptic species [7]–[11]. Most cryptic bat species, identified through molecular techniques, cannot be identified using external morphology but often do show other distinguishing characters, either in echolocation call [12]–[14] or more subtle morphological characters such as cranial morphology or tragus shape [15], [16].

The Rhinolophidae, or horseshoe bats, are restricted to the Old World. They are a taxonomically problematic group and the difficulties associated with resolving their taxonomy can be attributed to, amongst other things, a high level of morphological convergence [17], [18]. In addition to molecular techniques, echolocation calls have also contributed towards the identification of species or cryptic species. Echolocation frequency has been associated with partitioning of dietary resources and is thought to have a role in facilitating intraspecific communication, thus potentially allowing for species recognition and the discrimination of congeners [19]–[23].

Apart from the ecological factors responsible for driving the divergence amongst taxa, the genetic patterns of many species carry a signature of the effects of past climatic events on speciation [24], [25]. By using DNA data within a historical biogeographical framework, we can begin to improve our understanding of the evolutionary processes that drive diversification [6] and by linking the outcome hereof with ecological attributes, we can better explore the processes facilitating the observed diversification.

**Figure 1:**
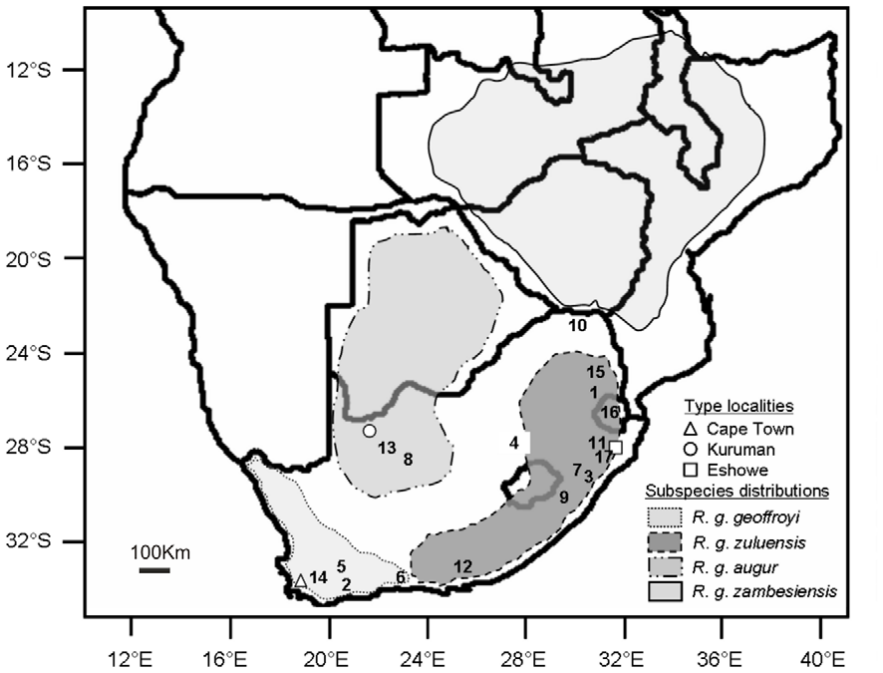
Rough distributions of the four *R. geoffroyi* subspecies as described in Roberts [**28**]. Type localities for three subspecies and the sampling sites used in this study are indicated where 1) Barberton, BT; 2) De Hoop, DHC; 3) Ferncliffe, FCC; 4) Winburg, FS; 5) Greyton, GREY; 6) Knysna, HKV; 7) Hopewell Farm, HWF; 8) Koegelbeen, KGB; 9) Kokstad, KSM; 10) Lajuma, LAJ; 11) Melmoth, MEL; 12) Maitland Mines, MM; 13) Postmasburg, POST; 14) Stellenbosch, STEL; 15) Sudwala, SUD; 16) Swaziland, SWZ; and 17) Yolland, YOL. The type locality for *R. g. zambesiensis* is only given as South Rhodesia, corresponding to present-day Zimbabwe.

*Rhinolophus clivosus* s.l. (type locality W. Arabia; [26]) is widespread throughout Arabia and Africa. It is predominantly a savanna woodland species, but is also found on forest fringes and in deserts [26]. This species/species-complex represents a taxon that is morphologically complex and variable [27]. Previously Roberts [28] recognized individuals from southern Africa, which were larger than *R. clivosus* individuals elsewhere in Africa, as an endemic species, *R. geoffroyi* (originally described by Smith 1829, type locality South Africa [26]). Within this species, he defined four subspecies on the basis of morphological and cranial characters and which were largely allopatric in distribution: 1) *R. g. geoffroyi*, 2) *R. g. augur*, 3) *R. g. zuluensis*, and 4) *R. g. zambesiensis* (rough distributions and type localities indicated in Fig. 1). However, *R. geoffroyi* is not currently recognized as a separate species because the name was unidentifiable and the type specimen apparently lost [26].

Here we assess the mtDNA genetic variation of *R. clivosus* s.l. in Africa, with particular focus on the South African representatives. Because four distinct subspecies were previously identified using morphology and cranial characters [28] we predict that there should be evidence of phylogeographic structuring of *R. clivosus* s.l in South Africa. Furthermore we assess the ecological diversity of these putative genetic groups by incorporating data on the echolocation and wing morphology of genotyped individuals and model the likely geographic ranges of the previously recognized subspecies using Maxent to gain insight into geographic factors that may underlie the genetic and ecological patterns observed. Due to the joint constraints of flight and detection of food in different habitats, echolocation and wing design have been assumed to form an adaptive complex [29]. If phylogeographic groups are closely linked to geographical attributes such as habitat, we predict that these groups should be largely allopatric in their distribution range and that they should clearly separate on echolocation and wing parameters.

## Methods

### Sampling, DNA Extraction and nucleotide sequencing

We followed international guidelines for the ethical treatment of animals and this research was approved by the ethics committee of Stellenbosch University (ID 2009B101003). Permits from AAA004-000400-0035(Western Cape), FAUNA 028/2010 and FAUNA 029/2010 (Northern Cape) and MPB 5254 (Mpumalanga) were obtained for the capture of bats. Tissue samples (mostly wing biopsy punches) from 144 individuals from various sampling localities (Table 1) were studied. Sequenced individuals included representatives from Egypt, Kenya, Mozambique, Namibia, South Africa, Swaziland and Tanzania. Within South Africa, we included samples that were collected from close to the type locality for three of the subspecies described by Roberts [28]: *R. g. geoffroyi*, *R. g. augur* and *R. g. zuluensis* (Fig. 1). Total genomic DNA was extracted using the DNeasy Blood and Tissue Kit (Qiagen, Hilden, Germany) following the manufacturer's recommendations.

**Table 1:** The number of samples (N) and haplotypes (n haplotypes) for each sampling locality.

Sampling locality	N	n haplotypes	Latitude	Longitude
Barberton (BT)	2	2	−25.72	31.27
De Hoop (DHC)	31	6	−34.43	20.42
Ferncliffe (FCC)	13	7	−29.55	30.32
Winburg (FS)	1	1	−28.54	27.05
Greyton (GREY)	1	1	−34.07	19.69
Knysna (HKV)	4	3	−33.95	23.17
Hopewell Farm (HWF)	14	6	−29.66	31.02
Koegelbeen (KGB)	5	1	−28.67	23.37
Kokstad (KSM)	3	3	−30.81	29.28
Lajuma (LAJ)	3	1	−23.03	29.43
Melmoth (MEL)	4	3	−28.59	31.40
Maitland Mines (MM)	2	1	−33.96	25.62
Postmasburg (POST)	11	2	−28.62	23.32
Stellenbosch (STEL)	1	1	−33.96	18.76
Sudwala (SUD)	29	9	−25.38	30.69
Swaziland (SWZ)	2	2	−25.96	31.17
Yolland (YOL)	3	3	−28.89	31.47
Egypt	1	1		
Kenya	5	1		
Mozambique	7	7		
Namibia	1	1		
Tanzania	1	1		

Geographical coordinates are not available for Egypt, Kenya, Mozambique, Namibia and Tanzania and these samples were not used in the population-level analyses.

A 456 bp section of the mitochondrial control region was amplified using the primers N777 and DLH1 [30], [31]. Polymerase chain reaction (PCR) thermal conditions were an initial 5 min denaturation at 95°C, followed by 35 cycles of 30 s at 94°C, 45 s annealing at 50°C, 45 s at 72°C, and a final extension cycle at 72°C for 10 min. All PCR reactions included a negative (all reagents, but no template) to control for contamination. A subsample of the PCR products was visualized on 1.0% agarose gels containing ethidium bromide. The remaining product was sent to the Core Sequencing Facility, Stellenbosch University, South Africa, where the PCR products were purified and cycle-sequenced using BigDye (Applied Biosystems, Perkin Elmer) chemistry. Sequencing products were then analysed on an ABI 3100 (Applied Biosystems, Perkin Elmer) automated sequencer. Chromatograms were visualized and aligned using BioEdit v7.0.1 [32]. Haplotypes are submitted under GenBank Accession numbers JN618191–JN618334. We included GenBank sequences (GQ220723, GQ220713, GQ220710) for three *R. ferrumequinum* individuals as the outgroup. *Rhinolophus ferrumequinum* is the sister species to *R. clivosus* [33].

### Phylogenetic analysis

Bayesian Inference (BI) was conducted in Mr Bayes 3.1.2 [34] with four simultaneous chains for three million generations, with parameters sampled every 1000 generations. Convergence of the MCMC chains was assessed by inspecting whether the standard deviation of split frequencies approached zero and the potential scale reduction factor (PSRF) reached 1.0 for all parameters. We also confirmed convergence using Tracer v 1.4.1 [35]. A 25% burnin was used and the 50% majority rule consensus tree was constructed from the remaining tree data. We used the TIM2 + I + G model of DNA substitution as suggested by jModelTest 0.1.1 [36] and Yule process as the tree prior. Uncorrected pairwise differences were calculated in PAUP* 4.0b10 [37] to allow for comparisons with other horseshoe bat species. Because our study involves the analysis of individuals from a single species, where both ancestral and derived hapotypes may coexist, we also constructed a minimum spanning haplotype network using TCS v1.21 [38] with a parsimony threshold set to 95%.

To estimate divergence dates analyses were conducted in BEAST 1.6 [35]. The Yule speciation process and a relaxed uncorrelated lognormal molecular clock model were selected. We used uniform tree priors with an upper limit of 5.3 million years ago (Mya) and a lower limit of 1.8 Mya. These dates were selected using the dates of fossils of the sister taxon *R. ferrumequinum* and members of the *R. ferrumequinum* group that were present from the early Pliocene (5.3 Mya) to the Pliocene-Pleistocene Boundary (1.8 Mya) [39]. The MCMC chain was run for 20 million generations, with parameters logged every 1000 generations. Results were evaluated using Tracer 1.4.1 [35]. The Effective Sample Size (ESS) values were >200 for all parameters, suggesting the MCMC run was sufficient and independent samples were incorporated to obtain valid parameter estimates [35]. Trees were collated using TreeAnnotator 1.6 where mean heights and a burin of 10% were selected.

### Genetic diversity and population structure

**Figure 2:**
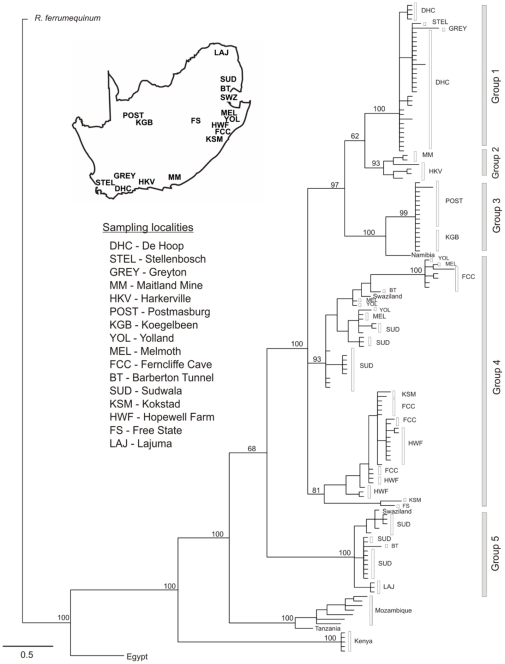
BI consensus topology. Values above nodes represent posterior probabilities. Grey bars indicate the five groups used in analyses of southern African bats. South African sampling localities are depicted graphically on the map.

From the outcomes of the above analyses, we grouped our sampling localities into five groups representing the main lineages for further analyses on the South African samples, including two specimens from Swaziland. Hereafter we refer to Groups 1 to 5 (see Fig. 2 tree) in the subsequent analyses and results. Standard molecular diversity indices, including haplotype and nucleotide diversity were calculated using DnaSP v 4.10.6 [40]. To assess the genetic structure within South Africa and the variation among groups, we used an Analysis of Molecular Variance (AMOVA) with 10 000 permutations implemented in Arlequin 3.5 [41]. We investigated genetic diversity over the landscape using graphical landscape interpolation plots in the program Alleles in Space [42]. Residual genetic distances and the midpoints of edges derived from Delaunay triangulation were used.

### Echolocation and wing morphology

Body mass (to nearest 0.5 g) of each captured bat was measured with a Pesola scale. Sex, age, and reproductive status of each bat were assessed. Only data for adult bats, identified by the fusion of the epiphyseal plates in the finger bones [43], were used to remove the confounding effects from physical immaturity.

Horseshoe bats are high duty-cycle echolocators and reduce echolocation call frequency in relation to flight speed to compensate for Doppler shifts [44]. To eliminate variation in frequency as a result of Doppler shift compensation, echolocation calls were recorded from handheld bats [45] to obtain their ‘resting frequency’ (RF). Calls were recorded using an Avisoft Ultrasound 116 bat detector (Avisoft Bioacoustics, Berlin, Germany) or Pettersson D980 bat detector (Pettersson Elektronik AB, Uppsala, Sweden) connected to an ASUS EEE 1005HA netbook (ASUSTek Computer Inc., Taiwan). The sampling frequency was set at 500 000 Hz (16 bits, mono), with a threshold of 16. The resultant wave files were analyzed with BatSound Pro (version 3.31b, Pettersson Elektronik AB, Uppsala, Sweden). Calls with a high signal-to-noise ratio, i.e. the signal from the bat was at least three times stronger than the background noise, were used in analyses. The dominant harmonic from each call was taken from the Fast Fourier Transform (FFT) power spectrum (size 512). A Hanning window was used to eliminate effects of background noise. Resting frequency (RF), the frequency of maximum energy in the constant frequency part of the pulse of a stationary bat, was measured from the power spectrum [46].

The extended right wing of each captured bat (after [47]) was photographed with an Olympus C730 digital camera (Olympus America Inc., New York, USA) and Canon G9 digital camera (Canon Inc., Japan) ensuring that the camera was positioned at 90° above the wing. The wing and the right hind limb and tail membrane was opened and secured in position to the graph paper with masking tape. We calibrated the wing images with the dimensions of the graph paper, and measured wingspan (to nearest 0.1 mm) and wing area (including body area without the head, and the area of the uropatagium; to nearest 0.1 mm^2^) using SigmaScan Pro 5 software (version 5.0.0, SPSS Inc., Aspire Software International, Leesburg, USA). These measurements were used to calculate aspect ratio (AR = b^2^/S where b is wingspan, m and S is wing area, m^2^) and wing loading (WL = M×9.81/S where 9.81 is the gravitational acceleration, m.s^−2^, and M is mass, kg; after [48]).

To determine whether echolocation and wing parameters could distinguish among the lineages revealed by the DNA analyses, we used a discriminant function analysis (DFA) on RF, WL and AR in Statistica 7 (Statsoft, Inc., Tulsa, Oklahoma).

### Ecological niche modelling

In light of the genetic and ecological diversity observed, the potential geographic ranges of the phylogeographic groups of southern African bats were modelled using Maxent v 3.1.0 [49], [50]. Maxent is a presence-background modelling technique that has performed well in recent tests [51], [52]. We used the sampling localities in this study and georeferenced locality data of *R. clivosus* s.l. in southern Africa based on 165 museum specimen records (see [53] for detailed information including museum accession number and latitude and longitude) to provide accurate presence data for the ecological niche models. Maxent can incorporate a “bias grid” with values that indicate relative sampling effort to address potential bias in sampling; however this requires a good understanding of the spatial pattern of the sample collection effort that produced the occurrence data. We did not produce such a bias grid because it is unlikely that there was more effort (by chance) in sampling a particular area of the region; our database consisted of records collected across the region by a number of researchers at different times with different sampling efforts and deposited in 12 museums. For us to introduce such a bias grid in the niche modelling would probably have introduced a sampling bias because of poorly informed sampling probabilities. *Rhinolophus clivosus* s.l. museum specimens were classified into four groups, based on the four subspecies and their putative distribution ranges previously recognized by Roberts [28]. We did not include specimens that occurred between Knysna and Maitland Mines (Group 2 bats) because we could not accurately assign them to one of the four subspecies due to the overlap in their distributions with both *R. g. geoffroyi* and *R. g. zuluensis*. To run the niche models, we used 11 bioclimatic predictors (annual mean temperature, isothermality, temperature seasonality, maximum temperature of the warmest month, minimum temperature of the coldest month, mean temperature of the wettest quarter, mean temperature of the driest quarter, annual precipitation, precipitation seasonality, precipitation of the warmest quarter, and precipitation of the driest quarter) derived from the WorldClim (http://www.worldclim.org). We used the World Wildlife Fund's Ecoregion database as a categorical variable [54], [55]. Ecoregions were defined as large land units that contain distinct assemblages of species and should approximate their natural distribution before anthropogenic land alteration. This data set was built on biogeography principles and with the collaboration of many researchers [54], and is a useful variable for natural land-cover across the region and for work with museum specimens that can be decades old [50]. We also used one topographic predictor (altitude) derived from a digital elevation model provided by the Shuttle Radar Topography Mission [56]. The environmental data were set to a spatial grid resolution of 2.5 arcminutes (∼5 km). The models were run with the convergence threshold set to 10^−5^, the maximum number of iterations set to 1000, the regularization multiplier set to 1, the output format set to logistic, and models were calibrated using both linear and quadratic features. We transformed the continuous probability output, ranging from 0 to 1, to a map representing the probability of suitable environmental conditions.

Model accuracy was evaluated with a threshold-independent receiver operating characteristic (ROC) analysis [50]. The area under the ROC curve (AUC) provides a single measure of model performance, independent of choice of threshold. Although the ROC and AUC have recently been criticised [57], [58], they can indicate the usefulness of the distribution models for identifying suitable areas of occurrence for species [51]. An AUC of 0.5 indicates that the model fits occurrence data no better than random predictions, while a value of 1 indicates perfect fit of predictions with data. AUC values >0.7 indicate useful predictions [59]. To test each model, 25% of the data in each run were randomly selected by Maxent and compared with the model output created with the remaining 75% of the presence data. The percentage contribution of each explanatory variable to model performance was evaluated with a jackknife procedure implemented in Maxent, where variables are successively omitted and then used in isolation to measure their relative and absolute contribution to the model.

## Results

### Phylogenetic analyses and genetic diversity

**Figure 3:**
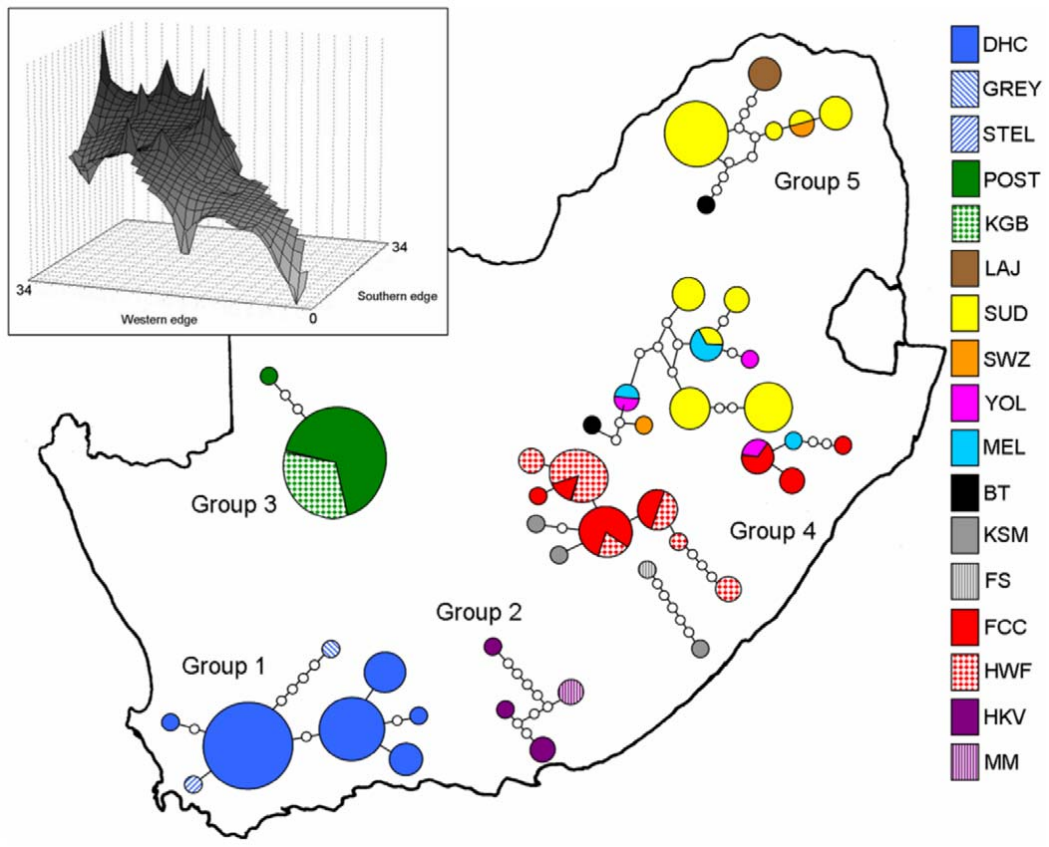
Minimum spanning network (95% threshold) for South African bats (including two individuals from Swaziland). Circle size represents haplotype frequency and patterns indicate the sampling locality for the proportion of individuals with that haplotype. Sampling locality codes are the same as in Figure 2 and Table 1. The inset shows the graphical interpolation-based representation of the genetic structure obtained over a South African landscape. Darker grey is indicative of high genetic diversity and lighter grey, of lower genetic diversity.

Bayesian analyses recovered six monophyletic major clades for the South African sampling localities (including Swaziland and Namibia) and three of these were supported by significant (≥95%) posterior probabilities (Fig. 2). When these major clades are overlaid onto sampling localities it is possible to identify five geographic groups comprising a Western Cape clade (Group 1), Knysna region clade (Group 2), Northern Cape clade (Group 3), a predominantly KwaZulu-Natal/Mpumalanga mixed group (Group 4) and a Mpumalanga/Limpopo Province clade (Group 5; Fig. 2). The geographic locations of these groups are shown in Figure 3. Outside of South Africa, the Egyptian sample diverged first from all other specimens sampled in this study followed by samples from Kenya, followed by individuals from Mozambique, and Tanzania. There is generally a strong correspondence between genetic divergence and geographic origin of samples. The phylogeographic position of the main groups in South Africa correspond geographically with the distributions of the previously described subspecies (Group 1 = *R. g. geoffroyi*; Group 3 = *R. g. augu*r; Group 4 = *R. g. zuluensis*; Fig. 2; [28]). Group 2 comprises individuals from Knysna and Maitland Mines near Port Elizabeth and based on geography could correspond with *R. g. geoffroyi* (for the Knysna samples) or *R. g. zuluensis* (for the Maitland Mine individuals). Group 5 comprises individuals from the most northern parts of South Africa, and may correspond with the fourth subspecies *R. g. zambesiensis*. At the higher level, sequence divergences indicate that the southern African representatives are as different from *R. clivosus* s.l. samples from further north in Africa (Kenya, Egypt) as they are from the sister species *R. ferrumequinum* (Table 2). Estimates for the time to most recent common ancestor, suggest that the South African individuals diverged from the individuals in Kenya, Tanzania, and Mozambique around 3.7 Mya (95% HPD: 1.447–4.076) and that all of the samples diverged from the most northern sample, Egypt, around 4.26 Mya (95% HPD: 2.23–5.29). Divergence of the five South African groups occurred during the late Pliocene and early Pleistocene (Table 3).

**Table 2:** Uncorrected pairwise distances (%) between *R. ferrumequinum* (RF) and *R. clivosus* s.l. from Egypt (EGY), Kenya (KEN), Tanzania (TAN), Mozambique (MOZ), Namibia (NAM), and the five South African groups (see Fig. 2. Bold, italic values in the diagonal indicate within group distances and hyphens indicate n = 1).

	RF	EGY	KEN	TAN	MOZ	NAM	Group 1	Group 2	Group 3	Group 4	Group 5
**RF**	***0.2–0.7***										
**EGY**	4.4–5.1	**-**									
**KEN**	12.2–12.4	11.6	***0***								
**TAN**	10.3–10.7	10.7	10.5	**-**							
**MOZ**	10.7–11.9	10.5–12.7	10.5–12.1	2.2–2.9	***0.7–8.3***						
**NAM**	11.7–12.1	12.1	11.6	6.8	7.7–8.6	**-**					
**Group 1**	11.6–13.0	11.2–11.8	10.3–11.0	7.5–8.8	7.2–9.9	4.2–5.0	***0.0–1.8***				
**Group 2**	11.8–13.2	11.2–12.3	10.8–11.0	8.1–8.6	8.6–10.1	4.4–4.8	2.6–3.3	***0.0–2.0***			
**Group 3**	11.8–12.3	11.8–12.5	11.6–12.1	7.2–7.9	8.1–8.6	2,2–2.9	3.5–4.8	3.7–4.6	***0.0–0.7***		
**Group 4**	10.5–13.8	9.9–12.7	9.9–11.9	6.6–8.3	7.0–9.6	3.7–5.5	3.7–6.4	3.3–6.6	5.0–6.8	***0.0–6.4***	
**Group 5**	9.6–11.0	10.7–11.4	9.2–11.7	8.6–8.9	8.1–9.6	7.7–7.9	7.2–8.3	7.0–8.1	8.1–8.3	6.1–9.0	***0.0–1.8***

**Table 3:** Molecular diversity indices for the five southern African groups showing samples size (N), number of haplotypes (nh), haplotype diversity (*h*) and nucleotide diversity (π).

	N	nh	*h*	π	tmrca	95% HPD
**Group 1**	33	8	0.767	0.004	2.07	0.654–2.553
**Group 2**	6	4	0.867	0.011	1.87	0.401–2.448
**Group 3**	16	2	0.125	0.001	1.54	0.327–1.793
**Group 4**	55	24	0.955	0.027	2.54	0.889–3.192
**Group 5**	19	6	0.749	0.749	2.31	0.611–3.129

Estimated dates of time to the most recent common ancestor (tmrca) means and 95% higher posterior densities provided in millions of years ago.

The deep intraspecific phylogeographic structure present in the South African samples is supported by the haplotype network (Fig. 3) where eight statistical groups were found. Four of the lineages (Groups 1, 2, 3 and 5) are confirmed monophyletic, and the fifth (Group 4) shows a considerable amount of variation (four groupings that could not be connected to each other with certainty and form part of the two lineages, with week posterior probabilities, in the BI analysis).

Nucleotide and haplotype diversity was the highest in Group 4 when compared with the other groups, whilst the lowest nucleotide and haplotype diversity was found in Group 3 (Table 3). A graphical interpolation-based representation of the genetic structure in South Africa indicates that the highest genetic diversity occurs in the north-eastern edge, corresponding with Groups 4 and 5 (Fig. 3 inset). AMOVA revealed significant genetic differentiation (all values p<0.0001) between the five geographic groups with 66.67% of the variation among groups, 19.72% among samples within groups and 13.61% within sampling sites (Φ_CT_ = 0.667; Φ_SC_ = 0.592; Φ_ST_ = 0.864). Pairwise Φ_ST_ values were high and significantly different between groups with the lowest values between Group 4 and the other groups (Table 4).

**Table 4:** Matrix of pairwise Φ_ST_ values between the five groups.

	Group 1	Group 2	Group 3	Group 4	Group 5
**Group 1**		p<0.0001	p<0.0001	p<0.0001	p<0.0001
**Group 2**	0.813		p<0.0001	p<0.0001	p<0.0001
**Group 3**	0.915	0.913		p<0.0001	p<0.0001
**Group 4**	0.653	0.498	0.679		p<0.0001
**Group 5**	0.93	0.893	0.948	0.724	

Φ_ST_ values are given below the diagonal and p values above the diagonal.

### Echolocation and wing morphology

**Figure 4:**
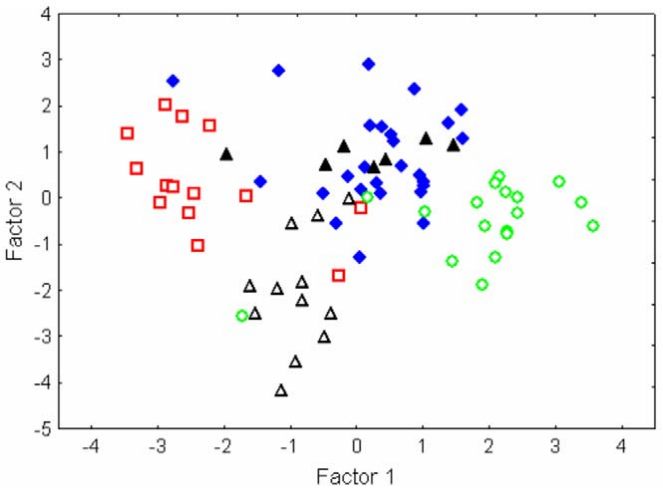
Plot of canonical scores from discriminant function analysis on echolocation and wing parameters. The five groups correspond to Figure 2 and squares = Group 3, open triangles = Group 1, closed triangles = Group 2, diamonds = Group 5 and circles = Group 4.

The five genetic groups were significantly separated by the DFA on wing (WL and AR) and echolocation (RF) parameters (Wilks' Lambda 0.148, F_(12, 185)_ = 16.35, p<0.001; Fig. 4, Tables 5 and 6). Four groups (excluding Group 2) were separated from each other along Function 1 (corresponding to resting frequency). Classification success for these four groups ranged from 75% (Group 1) to 89% (Group 4). Classification success for Group 2 was 0% and these bats were separated from Group 1 bats along Function 2 (corresponding to wing parameters). The above interpretation is supported by multivariate ANOVA comparing the wing and echolocation parameters among the five groups. Differences in AR, WL, and RF were found among all groups (F_(12, 172)_ = 12.55, p<0.001), but not between sexes (F_(3, 65)_ = 1.32, p = 0.275), although the interaction between sex and groups was significant (F_(12, 172)_ = 1.98, p = 0.029).

**Table 5:** Size, wing and echolocation parameters (mean ± SD; minimum–maximum) for the total number of bats in each of the five groups (see Fig. 2 tree).

Species group	N	Mass (g)	WS (cm)	WA (cm^2^)	WL (Nm^−2^)	AR	RF (kHz)
Group 1	21	20.2±1.37	34.5±1.11	197.3±14.67	10.1±0.66	6.1±0.63	92.5±0.52
	(m = 9; f = 12)	18–22.5	32.5–35.9	167.9–210.5	9.1–11.0	5.0–7.1	92.0–93.3
Group 2	7	16.9±1.31	33.7±0.01	206.4±16.0	8.0±0.32	5.5±0.38	92.2±0.55
	(m = 4; f = 3)	15.0–19.0	31.8–35.7	189.1–228.9	7.5–8.3	5.0–6.0	91.2–93
Group 3	14	19.6±1.62	35.0±1.31	212.5±11.35	9.0±0.7	5.8±0.47	91.0±0.91
	(m = 6; f = 8)	17.0–22.0	33.8–37.7	187.8–229.5	7.9–10.1	5.2–6.6	90.0–93.0
Group 4	18	16.2±1.25	33.4±0.01	197.8±11.12	8.4±0.71	5.6±0.36	93.8±0.53
	(m = 8; f = 10)	14.5–21.5	31.9–36.0	181.4–226.1	7.7–10.8	4.9–6.4	92.6–94.4
Group 5	27	15.6±1.25	32.4±0.01	190.8±14.48	8.0±0.74	5.5±0.28	92.4±0.69
	(m = 3; f = 24)	13.0–17.0	30.0–34.6	155.1–216.5	6.8–9.2	4.9–6.2	90–93

WS: wingspan, WA: wing area, WL: wing loading, AR: aspect ratio, RF: resting frequency. N  =  number of bats, where sample sizes for each sex (m =  males; f  =  females) are provided in parentheses below the total number of bats.

**Table 6:** Results of discriminant function analysis on echolocation and wing parameters of five groups.

	Function 1	Function 2	F to remove	Wilks' lambda	p
WL	−0.27	0.975	14.393	0.43	<0.001
AR	−0.416	0.186	2.452	0.169	0.054
RF	0.958	0.432	33.307	0.43	<0.001
Eigen value	2.122	1.161			
Cumulative %	64.6	99.9			
Wilks' lambda	0.148	0.463			
χ^2^	137.466	55.489			
d.f.	12	6			
p	<0.001	<0.001			

Groups correspond to Figure 2, WL  =  wing loading, AR  =  aspect ratio and RF  =  resting frequency.

The WL of Group 1 was significantly larger than those of the other groups (Tukey test p<0.01 in all cases), and the WLs of Group 2 and Group 5 were significantly smaller than those of Group 3 (Tukey test p<0.01 in both cases). Specifically, the WL of female Group 1 bats was significantly larger than those of male and female groups (Tukey test p<0.01 in all cases), except males from Groups 1 and 3 (Tukey test p>0.18 in both cases). The AR of Group 1 was significantly larger than those of Group 2, Group 4 and Group 5 (Tukey test p<0.05 in all cases). Specifically, the AR of female Group 1 bats was significantly larger than those of females in Groups 4 and 5 (Tukey test p<0.05 in both cases). The RF of bats in Group 4 was significantly higher than those of the other groups (Tukey test p<0.001 in all cases), and the RF of Group 3 was significantly lower than those of the other groups (Tukey test p<0.001 in all cases). Specifically, the RF of males in Group 4 was significantly higher than those of male and female groups (Tukey test p<0.05 in all cases), except females in Group 4. Similarly, the RFs of males and females in Group 3 were significantly lower than those of other male and female groups (Tukey test p<0.01), except males in Groups 1 and 5 and females in Group 2 (Tukey test p>0.5).

### Ecological niche modelling

**Figure 5:**
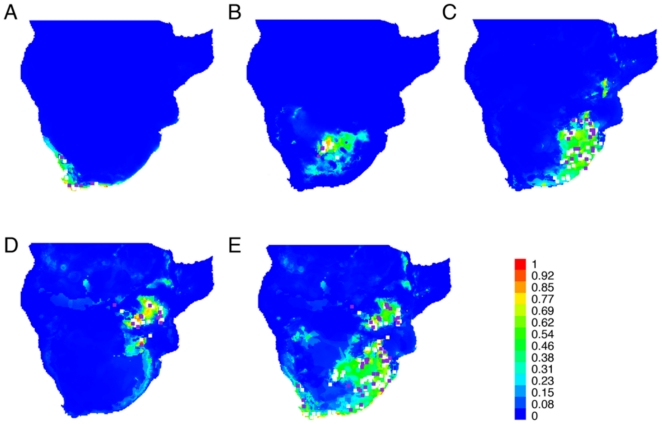
Ecological niche models (ENM). ENMs are based on occurrence records of four groups (see Fig. 2 tree) using current bioclimatic, altitude and ecoregions variables. A. Group 1, B. Group 3, C. Group 4, D. Group 5, E. all individuals. Shading shows probability of occurrence (i.e. habitat suitability) from low (blue) to optimal (red) conditions. Sampling locations used to build the ENM are shown as white squares and purple squares represent locations that were included in the training analyses.

Evaluation of model performance based on both training and test presence data indicated that the predictive ability of the models was higher than expected by chance (AUC>0.95 in all cases). The extent of the potential distribution ranges of Groups 1, 3, 4 and 5 (Fig. 5A–D) corresponded closely with the distribution ranges of the subspecies previously described by Roberts [28] (Fig. 1). To summarize the distribution of the four groups, we modelled the distribution of all individuals (Fig. 5E). It is notable that there was little overlap among the lineages. Jackknife tests of variable importance revealed that for three groups (Groups 1, 4 and 5) the environmental variable with the highest gain when used in isolation (i.e. appears to have the most useful information by itself) and the environmental variable that decreases the gain the most when omitted (i.e. appears to have the most information that is not present in other variables) was ecoregions. For the arid-adapted Group 3 this variable was the minimum temperature of the coldest month.

## Discussion

For many faunal species, their distributions coincide with the vegetation biomes, which in turn are influenced by temperature and rainfall and which would have experienced major changes during the Pliocene-Pleistocene. The impact that temperature changes had on the vegetation, may have contributed to diversification and speciation in many southern African taxa [60]–[64].

Sequence divergence values suggest that southern African *R. clivosus* s.l. bats are as genetically distinct from samples further north in Africa as from the sister species *R. ferrumequinum*, and the magnitude of the difference is similar to those between two horseshoe bat species from Kenya (10% for the mtDNA control region) - *R. eloquens* and *R. hildebrandtii* (Taylor et al. unpublished). The genetic distinctness of the southern African bats, currently recognised as *R. clivosus*, emphasises the need for a thorough taxonomic revision covering the entire distribution range of *R. clivosus* s.l. Within Africa, the large genetic differentiation between north-eastern and south-western lineages has been documented in other African vertebrate taxa [65]–[69] and has been linked to climatic changes during the Quaternary. In addition, samples from Mozambique show high levels of mtDNA genetic divergence both within Mozambique and between Mozambique and South Africa (Table 2). Recent investigations into other horseshoe bat species in Mozambique show that cryptic species are evident (Taylor et al. unpublished; Stoffberg et al. unpublished). The inclusion of nuclear markers and more comprehensive sampling of *R. clivosus* s.l bats throughout Mozambique, Namibia and Zimbabwe is required to fully resolve the taxonomic status of the lineages identified in this study and the geographic range of the southern African *R. clivosus* s.l..

Roberts [28] recognized the southern African representatives of *R. clivosus* s.l. as an endemic southern African species *R. geoffroyi*. Four of the lineages identified in this study do correspond to the geographical distributions of his proposed *R. geoffroyi* subspecies [28]: Group 1 bats (*R. g. geoffroyi*) in the Cape Floral Kingdom (CFK) that covers the extreme south-western and southern parts of South Africa, and where the climate is Mediterranean characterized by a winter rainfall season, Group 3 bats (*R. g. augur*) in the arid areas on the central plateau of the western half of the country, Group 4 bats (*R. g. zuluensis*) in the eastern, more mesic parts of South Africa, and Group 5 bats (*R. g. zambesiensis*), occurring in the northern parts of South Africa, consistent with many taxa showing distribution patterns that extend only into the northern-most parts of South Africa. These include birds (*Aquila ayresii, Coracias spatulatus, Falco dickinsoni, Melierax metabates, Myrmecocichla arnoti, Neafrapus boehmi, Poicephalus fuscicollis, Pytilia afra, Telacanthura ussheri, Telophorus nigrifrons*; [70]), reptiles (*Aparallactus lunulatus*; [71]), frogs (*Ptychadena uzungwensis*; [72]), and mammals (*Galago senegalensis, Heterohyrax brucei, Raphicerus sharpie, Rhabdomys dilectus dilectus, Cryptomys hottentotus nimrodi*; [73]–[75]), including bats (*R. fumigatus*, *R. landeri*, *Hipposiderus vittatus*, *Nycteris woodi*; [53]). Bats in Group 2 may represent a unique taxon that occurs in the Knysna Forest comprising patches of indigenous forest in the southeastern parts of the CFK.

Group 4 warrants additional research. Compared to the other groups, Group 4 comprises four discrete networks (Fig. 3), has the highest haplotype diversity and was not well recovered in the phylogeny with the two lineages comprising Group 4 having poor branch support (posterior probabilities <95%; Fig. 2). Although not well resolved, the two lineages recovered in the phylogeny may represent a predominantly ‘northern lineage’ and a predominantly ‘southern lineage’. The northern lineage includes samples from the northern parts of KwaZulu-Natal (e.g. YOL, MEL and some specimens from FCC; Fig. 2), Mpumalanga (e.g. SUD, BT; Fig. 2) and Swaziland, whilst the southern lineage includes bats from more southern locations in KwaZulu-Natal (e.g. FCC, HWF, KSM; Fig. 2) as well as from the Free State Province. These two lineages were assigned to Group 4 because their distributions correspond with the previously described subspecies *R. g. zuluensis*, the type locality being close to MEL and YOL sampling localities. However, on the basis of our results they may represent two separate genetic lineages that occur in sympatry in parts of their distribution and further geographic sampling will be required to assess the status of these two lineages. Future work should consider the inclusion of morphological and cranio-dental characters to assess the concordance between the molecular results found in this study and the characters originally used to define the subspecies.

The distributions of the five groups identified in this study are largely linked to the biomes present in South Africa today, similar to those reported for *Miniopterus natalensis* [60]. Furthermore, modelled distributions for bats assigned to one of the four previously recognized subspecies indicate that differences in ecoregions, as defined by the WWF, may play an important part in the present-day distribution patterns.

Pleistocene climatic cycling, with subsequent vegetation changes, has been shown to drive diversification in horseshoe bat species including *R. affinis* [76] and *R. ferrumequinum* [77] in East Asia. Estimates of divergence dates of the five groups suggest that they were present during the Quaternary (*ca* 2.6 Mya) a time when the climate was characterised by periodic glaciations and the more arid regions in the west of South Africa (Karoo biomes) had formed [78]. Climatic changes during the Pliocene/Pleistocene would have altered the suitable habitat in the mesic parts of the country through the fluctuations associated with the repeated expansion and contraction of savannas and woodlands/forests [78], resulting in bat populations becoming repeatedly isolated from one-another. During the Last Glacial Maximum (LGM *ca* 21–18 thousand years ago) the western arid regions of South Africa were dominated by desert [79] and many of the arid-adapted bats may have experienced population declines which could explain the low levels of haplotype and nucleotide diversity observed in Group 3 bats. The southern regions would have remained fynbos and shale renosterveld [64], [79], and may have harboured some individuals in suitable refugia such as the Cape Fold Mountains [80], [81]. The eastern interior of South Africa was characterised by steppe, with the xerophytic woodland/shrubland (including grassland) biome dominating the northern parts and extending southwards along the western border with the desert biome and on the eastern edge between the steppe and coast [79]. This habitat may have been more suitable for horseshoe bats along the eastern and northern sections of South Africa, providing more areas of refuge in which many bats could persist. Genetic diversity is also highest in these areas, and Lawes et al. [82] propose that major refugia for forest-associated taxa existed on the eastern coast of South Africa, from which they could have recolonised in a southerly or northerly direction. It may be possible that the two lineages in Group 4 (Fig. 2) reflect past recolonisation routes or migratory patterns during seasonal movements between winter and summer roosts that occur in a southerly or northerly direction. The inclusion of microsatellites in future analyses will help to assess levels of gene flow and investigate the potential for sex-biased dispersal.

Although divergence between the South African lineages occurred when much of the modern day biomes were already present, the continuous cycle of changing habitat followed by the survival of some individuals in refugia in their respective biomes, and subsequent recolonisation to suitable areas during more favourable conditions, may have reinforced the genetic divergence observed, and resulted in ecologically distinct groups. The combination of wing and echolocation parameters is important in determining where and how a bat can forage [29], [48], [83]–[86]. Size, echolocation call design, and wing morphology are part of the same adaptive complex allowing bats to utilize different habitats and prey [29]. Specifically, the short and broad wings, and high-duty-cycle echolocation, characterized by high frequencies, are adaptations for slow and manoeuvrable flight and detection of fluttering prey in structurally complex (“cluttered”) habitats [84]. A multivariate analysis of wing and echolocation characters differentiates between the five groups suggesting that they may be adapted to their local habitats. However, sample sizes for the groups are fairly small so differences in morphology and call frequency may reflect sub-sampling of geographically separate populations rather than selection for particular habitats. Hence, with increased sample size there may be a continuum of variation and increased overlap in echolocation and wing morphology rather than discrete states. Moreover, the variation in echolocation and wing parameters was found to be as large within groups as between groups, thus differences in these parameters among groups are unlikely to provide differential access to prey and/or habitat.

Another possible explanation for the separation of bats along Function 1 of the DFA is that the differences in echolocation frequency may facilitate communication amongst conspecifics [20], [23] and assist in distinguishing amongst heterospecifics even when there is overlap of species' frequency bands [22]. In this regard, it is perhaps notable that along Function 1 the Knysna bats (Group 2) show the most overlap, particularly with bats from Groups 1 and 5. Knysna bats may not need unique frequency bands to distinguish conspecifics because they are unlikely to encounter bats from Group 5 based on geographic distribution, and are separated from bats of Group 1 on the basis of their wing morphology. Specifically, their wing loading was significantly lower than that of bats in Group 1. Lower wing loadings are more suitable for flying in structurally complex habitats and the differences observed may be due to the selection pressures exerted by the structural complexity of the Knysna forests where these bats occur as opposed to the Fynbos habitats of the Group 1 bats. Nonetheless, too much overlap of species' frequency bands should be selected against if the trait is important in social interactions. Future studies should investigate if horseshoe bats from different geographic locations can differentiate each other despite overlap in call frequencies or if the bats are using other call parameters such as shape of call or social calls for the recognition of conspecifics. Alternatively, initial differences in frequency may be due to founder effects and these differences in frequency may be maintained in populations because vertical transmission from mother to offspring plays a role in the fine-tuning of frequency in horseshoe bats [87]. Conversely, it is unlikely that call differences among groups can be explained by differences in humidity. Humidity influences the degree to which atmospheric attenuation affects echolocation calls. High frequencies are particularly subject to rapid atmospheric attenuation, and attenuate even more rapidly under humid conditions. According to this hypothesis therefore selection should favour lower frequencies in bats that occur in more humid areas (i.e. Groups 4 and 5). However, these bats used the highest frequencies.

Genetic structure correlated with distinct ecological attributes as defined by the separation of the groups using echolocation calls and wing morphology has been argued to suggest incipient speciation [60]. The strong concordance between genetic diversity and ecological diversity suggests that the five geographic groups examined in this study are distinct and are adapted to their respective habitats, and should be considered as separate units in conservation planning. However, there are limitations to the inferences that can be made using a single marker that evolves quickly and is maternally inherited (in this study, the mtDNA control region). It is possible that these distinct groups may reflect species-level or subspecies splits, or that they are population level effects and further analysis is required to differentiate between these hypotheses. Also, genetic introgression has been shown in other horseshoe bat species [88] and the inclusion of nuclear markers will be required to test for introgression among southern African *R. clivosus* s.l. lineages. Thus further research incorporating multilocus DNA sequence data, and more traditional taxonomic characters such as skull morphology, will be required to assess the taxonomic status of these lineages in South Africa and assist in resolving the taxonomic status of *R. clivosus* s.l. in southern Africa.
